# Take Me to (the Empty) Church? Social Networks, Loneliness and Religious Attendance in Young Polish Adults During the COVID-19 Pandemic

**DOI:** 10.1007/s10943-021-01486-1

**Published:** 2022-01-18

**Authors:** Ł. Okruszek, A. Piejka, K. Żurek

**Affiliations:** Social Neuroscience Lab, Institute of Psychology, Polish Academy of Sciences, 1 Jaracza Street, 00-387 Warsaw, Poland

**Keywords:** Religious attendance, Social networks, Loneliness

## Abstract

A significant body of research supports the relationship between religious attendance, objective and subjective social networks characteristics, and mental well-being. This trajectory may be particularly important in the wake of the COVID-19 crisis. Thus, the current study examined the relationship between religious attendance, social network characteristics, loneliness, and mental well-being in a sample of 564 young adults (aged 18–35 years) soon after the first COVID-19-related restrictions were imposed in Poland. In line with previous findings, both frequent (FAs) and infrequent religious attenders (IAs) reported more people in their social networks compared to non-attenders (NAs). Further analysis revealed full mediation of religious attendance (FAs vs. NAs) via social network size on loneliness and mental well-being. This pattern of results was still observed after the exclusion of worship-based affiliates from the social network score. A follow-up survey carried out one year later (*N* = 94) showed that all three groups of participants (FAs, IAs, and NAs) reported increased loneliness and decreased mental well-being. Taken together, these findings show that the influence of religious attendance on social functioning cannot be attributed solely to congregational relationships.

## Introduction

For decades now, a growing body of work has provided evidence for positive associations between religiosity and physical and mental health. Several meta-analyses have corroborated the robust link between religious involvement and mortality (Chida et al., 2009; Lutgendorf et al., 2004; McCullough et al., 2000). Religion and spirituality have been related to important markers of cardiovascular health (e.g., blood pressure, cholesterol, markers of myocardial infarction), inflammation, and immunity (e.g., C-reactive protein and viral infections; Shattuck & Muehlenbein, 2020). The impact of religion has been found to be similar to or even stronger than many recommended health interventions (Lucchetti et al., 2011). Moreover, a recent meta-analysis (Garssen et al., 2021) found a small effect of religiosity (i.e., participation in public religious activities and importance of religion) on mental health. Investigations of religious involvement and its association to multiple aspects of well-being are particularly crucial during crises such as the COVID-19 pandemic, which can pose a severe threat to both physical and mental health.

While a plethora of evidence supports the link between religiosity and health, the diversity of possible outcomes related to different aspects and types of religious involvement constitute a challenge to empirical research on the religion-health association (Park et al., 2017). Multiple potential mediators (e.g., hope, optimism, purpose and meaning, adaptation to and coping with bereavement, self-esteem and self-efficacy; for a review see Seybold, 2007) have been discussed as factors underlying the relationship between religiosity and mental and physical health.

Notably, it has been argued that one of the most important aspects of religion in the context of its effect on well-being is social integration and increased involvement with both formal and informal social networks (Fraser et al., 1997; Hayward & Krause, 2014). Religiosity usually involves some sort of social activity and more or less regular meetings with co-believers. For example, people who declared to be active in their faith communities received various types of religious social support (Le et al., 2016). Moreover, receiving emotional support from other church members is positively correlated with an increased sense of belonging to the congregation, which, in turn, is linked to higher satisfaction with one’s health (Krause & Wulff, 2005). At the same time, a meta‐analysis conducted by Shor and Roelfs (2013) revealed no significant differences for the mortality hazard ratio between religious and nonreligious participation, suggesting that the positive health results of religious involvement are mostly due to its social component. In addition, a study investigating the links between religious disaffiliation and well-being found that individuals who disaffiliate but maintain regular attendance showed no health or well-being disadvantages compared to those consistently affiliated (Fenelon & Danielsen, 2016). These results support the notion that organized religious attendance serves a similar evolutionary role with organized bonding events (Dunbar, 2021), and thus their function is primarily social.

While a large body of research points toward the beneficial effects of social integration or increased social support on well-being outcomes and physical health in religious attenders, these effects may be even further enhanced by subjective appraisals of one’s social relationships. Loneliness or perceived social isolation (PSI), defined as a subjective perception of insufficient quality or quantity of one’s social bonds, may be observed even in the absence of objective social isolation (OSI), manifested in a lack of social activities and a minimal number of social ties.

Although often used interchangeably, OSI and PSI may be seen as two overlapping, yet non-synonymous phenomena (Coyle & Dugan, 2012). Large cohort studies have shown the association between OSI and PSI both in young (Ge et al., 2017; Matthews et al., 2016) and older adults (Coyle & Dugan, 2012). Yet, in most of the studies, the correlation between the two constructs is moderate. This latter observation is congruent with the view that objective social functioning may be one of the drivers of loneliness; however, the relationship is not deterministic.

Furthermore, a mounting body of evidence has shown that both OSI and PSI may have detrimental effects on physical and mental health, and should be treated as significant public health risk factors (Holt-Lunstad et al., 2015). A meta-analytic review by Holt-Lunstad et al. (2015) found that both of these factors have a similar impact on early mortality risk (29% and 26%, respectively). OSI and PSI are also related to cardiovascular disease (Holt-Lunstad and Smith, 2016; Valtorta et al., 2016) and inflammation (Smith et al., 2020), and both constructs can have a synergistic effect on mortality (Beller & Wagner, 2018). Another overview of systematic reviews identified significant associations between OSI, PSI, and increased all-cause mortality and poorer mental health outcomes (Leigh-Hunt et al., 2017) Moreover, longitudinal studies have shown that loneliness can be a predictor of the onset of common mental disorders (Nuyen et al., 2020) and poorer depression outcomes (Wang et al., 2018). It can also mediate the relationship between childhood trauma and the development of psychopathology later in life (Shevlin et al., 2015). Loneliness is also strongly and negatively linked to subjective well-being (VanderWeele et al., 2012), and positively linked to daily stress (Donae & Adam, 2010).

Notably, these effects are in contrast to the positive effects of religiosity on health that have been reported in the literature. Given the social aspects of the majority of religious movements, the relationship between religious involvement and both objective and subjective social functioning can have important consequences for one’s well-being.

Knowing that the negative effects of loneliness and social isolation on health outcomes may be synergistic (Beller & Wagner, 2018), it is worth investigating the relationships between involvement in religious activity, objective social network characteristics, and subjective appraisals of one’s social functioning. In line with this notion, a large US study with a representative sample of older adults (aged 57–85 years) observed that the effects of involvement in religious institutions on reduced loneliness in later life are mediated by increased social integration and social support in frequent attenders (Rote et al., 2013). Similarly, a seminal study by Ellison and George (1994) found that frequent churchgoers from an American Southeastern community sample reported larger social networks and more favorable perceptions of the quality of their social relationships compared to counterparts who did not attend church.

While these findings highlight the beneficial social outcomes of religiosity, it remains unclear whether these effects are driven directly by increased contact with other members of a religious group, or whether the improved social functioning of religious attenders extrapolates to other, secular social environments. While the literature on the subject often focuses on the former (i.e., support from fellow believers; Krause & Wulff, 2005), a growing body of research emphasizes the more general social impact of religious affiliation. For example, Lewis et al. (2013) provided evidence that the effects of church attendance may proliferate on other secular domains including civic and neighborly activities via the strong network of religious friends. Ten Kate et al. (2017) showed that the link between religious affiliation and life satisfaction is strictly dependent on the more general social context (e.g., the social position of one’s religious group and embeddedness in non-religious social structures). Moreover, Hastings (2016) found that people who are spiritual but not religious do not differ in their social connectedness levels from religious attenders, and that more general, informal social networks are more important than memberships in formal religious structures in improving social life. Thus, when studying the links between religiosity, social functioning, and well-being, it is crucial to examine the broader effects that religiosity can have on various aspects of one’s social network and not only those expressly linked to the direct effects of belonging to religious groups.

Finally, while the relationships between social networks, loneliness, and well-being have been extensively studied, this line of research has been focused mostly on older adults (Rote et al., 2013), and even the more general studies have tended to oversample older individuals (Ellison & George, 1994). Yet, both the religious practices (Ainlay et al., 1992; Argue et al., 1999) and social network characteristics (Green et al., 2001) of young adults deviate significantly from the patterns observed in older populations. Moreover, it is the young adults who are found to be the most vulnerable to the detrimental effects of the current social restrictions imposed due to the COVID-19 pandemic (Beam & Kim, 2020) and at risk of loneliness in general (Barreto et al., 2021). Thus, it is of particular importance to investigate the extent that religious attendance can impact social functioning in this group, especially in the context of forced constraints put onto people’s direct interactions with others and the disruption of their daily social routines.

Thus, the general aim of the current study is to examine the relationships between religious attendance, objective social network characteristics, loneliness, and mental well-being in a cohort of young individuals. Our first set of hypotheses is linked to the between-group differences between religious attenders and non-attenders (NAs) with regard to the objective and subjective characteristics of their social functioning. Namely, we hypothesize that:

### *H1*

Frequent attenders (FAs) will have larger overall social networks compared to NAs.

### *H2*

FAs will report less loneliness compared to NAs.

### *H3*

FAs will have larger social networks, compared to NAs, even after excluding contacts stemming from religious-based networks.

Furthermore, we plan to supplement the confirmatory analyses listed above with the investigation of between-group differences with regard to: 1) specific types of social networks, including specific family-based networks (partner/children, parents/in-laws, distant relatives), close friends, and other networks (e.g., religious-based networks, university/work networks), and 2) specific types of loneliness (loneliness in intimate relationships, loneliness in casual social networks, lack of a sense of belonging to a larger group or community).

The second main aim of the current study is to examine the relationships between religious attendance, objective characteristics of one’s social networks, subjective appraisals of one’s social networks, and one’s mental well-being. Particularly, we hypothesize that:

### *H4*

The positive impact of religious attendance on mental well-being is double-mediated by 1) the larger size of one’s social network, and 2) a lower level of loneliness.

Furthermore, we expect this effect to be independent of the inclusion of contacts stemming from religious-based networks in the social network size.

Finally, the third aim of the study is to investigate the impact of COVID-19 restrictions on loneliness and mental well-being in religious attenders and NAs. Namely, as church services were relatively unaffected by COVID-19 lockdown restrictions in Poland and religious attenders could participate in ongoing religious services throughout the period of COVID-19 restrictions, we hypothesize that:

### *H5*

Religious attenders will show less of an increase in loneliness during the follow-up examination as compared to NAs.

## Methods

### Procedure

The first part of the study was performed between March 15 and 20, 2020 via the Qualtrics questionnaire platform. Volunteer participants were recruited on social media platforms. At the beginning of the survey all of the participants were informed that all the data will be anonymized, analyzed on the group level and gathered only for research purposes, and that the participation withdrawal is possible at any moment of the survey. The examination was part of a larger project investigating the associations between social functioning and the COVID-19 response (the protocol of the study was approved by the Ethical Committee at the Institute of Psychology, Polish Academy of Sciences).

The final sample consisted of 564 participants aged 18–35 years. Religious attendance was assessed via a question about the frequency of participation in religious services (reflecting the situation during data collection): ‘Besides the special occasions (weddings, funerals, etc.), how often do you participate in Masses, church services and religious meetings?’. Three categories linked with regular attendance (more than once a week/ once a week/ once—twice a month) were pooled to create a FAs group. Two categories linked to infrequent attendance (only during religious holidays/ less frequent than in previous options) were pooled to create infrequent attenders (IAs). Finally, the NAs group was created from participants who chose the response ‘Never’.

The characteristics of each subgroup (148 FAs, 205 IAs, 211 NAs) are shown in Table 1. No between-group differences were observed for age [*F*(2, 563) = 0.91, *p* = 0.402], sex [χ^2^(2) = 5.71, *p* = 0.058], education [χ^2^(16) = 12.90, *p* = 0.680], current employment status [χ^2^(2) = 2.55, *p* = 0.280] or current student status [χ^2^(4) = 2.19, *p* = 0.702] between the three groups. However, significant differences between groups were observed for the size of place of origin [χ^2^(6) = 61.78, *p* < 0.001] and current living place [χ^2^(6) = 22.08, *p* = 0.001]. In both cases, the most pronounced differences were observed for the fraction of participants coming from large (over 500 thousand inhabitants) cities (more prevalent in NAs compared to FAs) and for the fraction of participants from rural regions (more prevalent in FAs compared to NAs).

**Table 1:** Detailed information on the participants

Variable	Whole sample	Religious attendance		
		Frequent attenders	Infrequent attenders	Non-attenders
Age (years)				
* M*	26.41	22.97	23.44	23.46
* SD*	9.24	3.3	3.85	3.74
Sex (% female)	78.1	85.8	81.5	75.8
Student status (%)	73.1	84.5	79	59.2
Place of origin (%)				
Village	16.8	35.1	15.7	8.1
Town < 50 k	19.9	24.3	22.1	15.2
City 50–500 k	23.3	15.5	27.5	27
City > 500 k	39.4	25	34.8	49.8`
Current place of living (%)				
Village	9.8	17.6	6.4	5.7
Town < 50 k	8.4	9.5	7.8	6.6
City 50–500 k	10.4	10.8	11.8	8.1
City > 500 k	70.4	62.2	73	79.6
Civil status (%)				
Married / in marital-like relarionship	50.5	44.5	56.1	48.4
Not married / in marital-like relationship ever	36	41	32.9	35.2
Separated	.6	1.2	.8	-
Divorced / currently single	11.8	11	9.3	14.8
Widowed	1.5	2.3	.8	1.6

During the first wave of the study, 271 participants agreed to be contacted for further follow-ups. Among those participants, 94 (35%) completed a follow-up survey including the R-UCLA and GHQ (but not the SNI) between March 15 and April 1, 2021. The follow-up groups did not differ in terms of age [*F*(2, 91) = 0.30, *p* = 0.741] or sex [χ^2^(2) = 1.99, *p* = 0.370]. However, it is worth noting that the follow-up was completed mostly by NAs (*n* = 38) and IAs (*n* = 34), while a relatively small group of FAs (*n* = 22) participated in this stage of the study.

#### PSI Measurement

The Revised UCLA Loneliness Scale (R-UCLA; Kwiatkowska et al., 2017) in Polish was used to assess PSI. This self-report scale includes 20 items formulated as declarative sentences referring to the level of satisfaction in interpersonal relationships and provides the results on three subscales: Intimate Others, Social Others, and Belonging and Affiliation. The original R-UCLA version has a good internal consistency (alpha range from 0.89 to 0.94; in the current study: alpha = 0.92), test–retest reliability (*r* = 0.73 over 1-year time), and is correlated with different measures of loneliness and social support assessment (Russell, 1996). The Polish version of the R-UCLA has also proven to be highly reliable (ω_Total Score_ = 0.92) and to have good external validity, including correlations with measures of self-esteem and shyness (Kwiatkowska et al., 2017).

#### Social Network Measurement

Social network size was measured through the self-report Social Network Index (SNI) translated to Polish. This questionnaire is aimed at estimating the number of social environments in which the respondent has regular contact with others. The following categories are included in the SNI: partner (including marital-like relationships), children, parents, in-laws, relatives other than partner, parents and children, close friends, affiliates from church, temple or other religious groups, teachers and co-students, co-workers, volunteering societies, and other groups. Each category starts with a question on having a particular type of relative or participation in a particular activity (respectively: number of children, living parents, living in-laws, number of close relatives, number of close friends, belonging to a religious group, regular attendance at educational courses, employment status and number of work subordinates, engagement in voluntary work, other group membership) followed by a question on the number of people within the given category that the respondent has regular contact with at least once every two weeks. Additionally, the SNI questionnaire begins with an item inquiring about the respondent’s marital status and, at the end, the subject is asked to list other groups along with the numbers of other members regularly contacted.

The Polish translation of the SNI was created after obtaining approval from the author of the original version of the scale (Cohen et al., 1997).

#### General Health Questionnaire (GHQ)

Mental well-being was assessed via the Polish version of the 30-item GHQ (Frydecka et al., 2010). This questionnaire has been shown to have very high reliability (Cronbach’s alpha = 0.97; in the current study: alpha = 0.93) and provides scores on three dimensions: Anxiety and Depression, Interpersonal Relationships, and General Functioning. Higher overall GHQ scores are associated with greater psychological symptoms and thus lower mental well-being.

#### Situational COVID-19-Related Social Variables

As the data for the study were collected soon after the first COVID-19 safety measures were imposed, we asked two specific questions regarding the participants’ social surroundings in the wake of COVID-19 restrictions. First, the participants were asked with whom they were planning to spend the subsequent two weeks, as the ‘stay at home’ recommendation was just publicly announced. Participants were given a list of possible options (Partner/ Children/ Parents/ Siblings/ Other family members/ Unrelated roommates/ Strangers/ Other). The participants were also asked to identify whom they could ask for help in case of being quarantined (Nobody/ Partner/ Family/ Neighbors/ Friends/ Co-workers/ Public services/ Other). In the case of choosing the ‘Other’ option, participants were further asked to manually enter the details of their response.

### Statistical Analysis

A between-group ANOVA was conducted with 3-level religious attendance as an independent variable and the number of people in social networks as indicated by the sum score in the SNI. All the post-hoc comparisons were Tukey corrected at *p* = 0.05.

The same approach was used to investigate the between-group differences for specific SNI sub-scores, with an exception for relationship status, which, in line with the SNI scoring guidelines, is recoded into a dichotomous variable to indicate whether the participant is married or in a marital-like relationship. Thus, relationship status was analyzed using the chi-squared test. Finally, a between-group ANOVA was used to analyze whether the effects of religious attendance could be observed after excluding worship-based affiliates from the general SNI score (‘adjusted SNI score’).

Similarly, the R-UCLA general score and subscale scores, as well as the GHQ general score, and the number of people with whom participants were spending the subsequent 2 weeks, were analyzed using between-group ANOVAs. The remaining responses to the COVID-19 situational variables were analyzed using the chi-squared test.

Mediation analyses were conducted using dummy variables coding with NAs as a reference variable, thus producing two categorical variables (IAs vs. NAs, FAs vs. NAs). First, to replicate Rote et al.’s (2012) findings, we examined whether the effects of religious attendance on loneliness were mediated by social network size. Next, we extended the mediation model by testing the double mediation of religious attendance on mental well-being via social network size and loneliness. Both simple and serial mediation were analyzed using either the full SNI score or the SNI score without worship-based affiliates. Mediation analyses were performed using PROCESS 3.5 with 10,000 bootstrap samples for bootstrap confidence intervals (Hayes, 2017).

Finally, to investigate the longitudinal changes in loneliness and mental well-being, we used repeated measures ANOVA with the between-group factor religious attendance (3 levels: NAs/ IAs/ FAs) and the within-group factor time (2020 vs. 2021).

## Results

### Religious Attendance and Social Networks

In line with previous research, religious attendance was linked to the objective characteristics of participants’ social networks, with significant effects observed for social network size [*F*(2, 561) = 11.98, *p* < 0.001]. Both FAs (20.03 ± 10.18) and IAs (16.85 ± 8.81) had larger social networks compared to NAs (15.29 ± 8.49; vs. IAs: *p* = 0.004; vs. FAs: *p* < 0.001), which is in line with H1.

When particular types of the relationships were investigated, we observed differences for relationship status [χ^2^(2) = 7.74, *p* = 0.021; in marital-like relationship: 48.3% of NAs, 56.1% of IAs, 41.2% of FAs], relatives other than partner, parents and children [*F*(2, 561) = 12.64, *p* < 0.001; more in FAs (2.25 ± 2.05, p < 0.001) and IAs (1.72 ± 1.75, p = 0.012) than in NAs (1.32 ± 1.41)], and co-students or teachers [*F*(2, 561) = 4.98, *p* = 0.007; FAs (4.58 ± 2.84) > NAs (3.60 ± 2.96) at *p* = 0.005]. Finally, a significant between-group difference was observed for the number of affiliates from church, temple, or other religious groups [*F*(2, 561) = 73.39, *p* < 0.001], with both FAs (1.68 ± 2.60) and IAs (0.12 ± 0.73) reporting more such affiliates than NAs at *p* < 0.001.

As we expected (H3), the difference in the overall size of the social network was still significant after excluding members of church or religious groups from the overall score [*F*(2, 561) = 5.41, *p* = 0.005]. Further analysis of this effect revealed that, in case of the adjusted social network size, only the difference between FAs (18.34 ± 8.99) and NAs (15.29 ± 8.49) was significant (*p* = 0.003), while IAs (16.73 ± 8.67) did not differ from any of the remaining groups.

### Loneliness

No significant differences were observed between the groups in the mean level of loneliness [*F*(2, 561) = 2.61, *p* = 0.074]. However, when the R-UCLA subscales were analyzed, no between-group differences for the R-UCLA Intimate Others subscale [*F*(2, 561) = 1.23, *p* = 0.293] were observed, but FAs had lower levels of R-UCLA Social Others [*F*(2, 561) = 3.93, *p* = 0.020] and R-UCLA Belonging and Affiliation [*F*(2, 561) = 3.07, *p* = 0.047] than NAs. These findings are partially in line with our expectations of lower loneliness levels in FAs comparing to NAs (H2).

### Mental Well-Being

No significant differences were observed between the groups in the level of mental well-being [*F*(2, 561) = 1.94, *p* = 0.144].

### Mediation Analysis

Frequent religious attendance compared to non-attendance [*F*(2, 561) = 11.98, *R*^2^ = 0.041, *p* < 0.001; FAs vs. NAs: beta = 0.512, *t* = 4.87, *p* < 0.001] was a significant predictor of social network size. However, no similar effect was observed for infrequent religious attendance vs. non-attendance (IAs vs. NAs: beta = 0.169, *t* = 1.76, *p* = 0.08).

Social network size [*F*(3, 560) = 22.57, *R*^2^ = 0.11, *p* < 0.001; beta = − 0.321, *t* = − 7.87, *p* < 0.001], but neither IAs vs. NAs (beta = − 0.112, *t* = − 1.20, *p* = 0.23) nor FAs vs. NAs (beta = − 0.064, *t* = − 0.61, *p* = 0.54), predicted the loneliness level.

The indirect effect (full mediation) of the FAs vs. NAs on loneliness via social network size [beta = − 0.164, *CI* = (− 0.251; − 0.090)] was found to be significant.

Next, the same analysis was repeated for the adjusted SNI score. Again, FAs vs. NAs [*F*(2, 561) = 5.41, *R*^2^ = 0.019, *p* < 0.001; beta = 0.349, *t* = 3.28, *p* < 0.01], but not IAs vs. NAs (beta = 0.165, *t* = 1.69, *p* = 0.091), predicted adjusted social network size. Further, loneliness was predicted by adjusted social network size [*F*(3, 560) = 23.68, *R*^2^ = 0.11, *p* < 0.001; beta = − 0.325, *t* = − 8.076, *p* < 0.001], but not by the FAs vs. NAs (beta = − 0.115, *t* = − 1.12, *p* = 0.262) or IAs vs. NAs (beta = − 0.113, *t* = − 1.21, *p* = 0.226).

Again, the analysis revealed a significant indirect effect (full mediation) of the FAs vs. NAs on loneliness via adjusted social network size [beta = − 0.113, *CI* = (− 0.193; − 0.044)] (findings in line with H4).

Finally, both of the mediation analyses described above were extended by adding the overall well-being score as an outcome measure. Loneliness level was a significant predictor of overall mental well-being [*F*(2, 561) = 30.54, *p* < 0.001; R^2^ = 18, beta = 0.400, *t* = 9.86, *p* < 0.001]; however neither SNI (beta = − 0.048, *t* = − 1.17, *p* = 0.244) nor any of the religious attendance variables (IAs vs. NAs: beta = 0.034, *t* = 0.381, *p* = 0.704; FAs vs. NAs: beta = − 0.088, *t* = − 0.888, *p* = 0.375) predicted mental well-being. The same pattern of results was observed when the adjusted SNI score was included instead of the full SNI score.

**Fig. 1:**
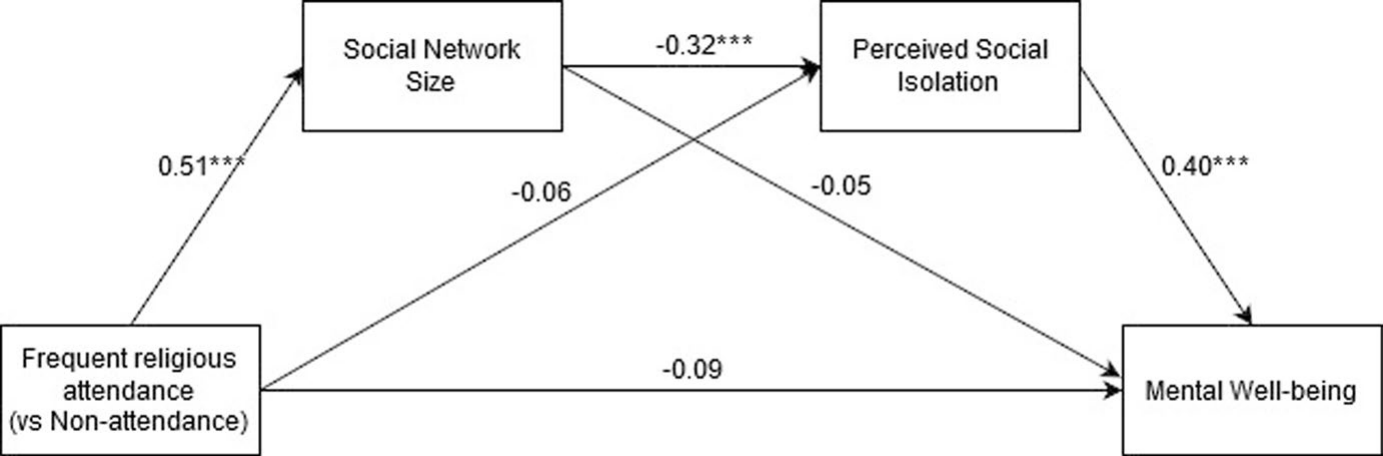
The results of the double mediation model for frequent religious attendance (vs. non-attendance). Social Network Size represents the full SNI score. *-*p* < .05, **-*p* < .01; ***-*p* < .001

In the case of both the full and adjusted SNI scores, the double mediation of the religious attendance FAs vs. NAs category via social network size and loneliness on mental well-being was observed [full SNI: beta = − 0.066, *CI* = (- 0.104; − 0.035); adjusted SNI: beta = − 0.045, *CI* = (- 0.080; − 0.017)]. In both cases no direct effects, or simple mediation (via SNI or R-UCLA) were significant. The model including FA (vs. NA) and the full SNI scores as predictors is presented in Fig. 1.

### Lockdown Physical Contact and Social Resources

A main effect of religious attendance on the number of people with whom the participants were predicting to spend the next 2 weeks was observed [*F* (2, 561) = 8.02, *p* < 0.001], with FAs spending the subsequent 2 weeks with more people compared to NAs (*p* < 0.001). Investigation of the specific types of relationships showed that FAs were less likely to spend the next two weeks with a partner (FAs: 17.5%/ IAs: 35.6%/ NAs: 32.7%; χ^2^(2) = 14.75, *p* = 0.001) compared to other groups. At the same time, FAs were more likely to spend the next two weeks with parents (FAs: 68.2%/ IAs: 56.1%/ NAs: 49.3%; χ^2^(2) = 12.79, *p* = 0.002) and siblings (FAs: 46.6%/ IAs: 32.2%/ NAs: 28.9%; χ^2^(2) = 12.96, *p* = 0.002). No significant effects were found for the remaining categories (children, other family, roommates, coworkers, strangers).

When COVID-19-related social resources were investigated, the only significant effect was observed for less FAs expecting possible help from partners compared to other groups (FAs: 31.1%/ IAs: 47.3%/ NAs: 41.2%; χ^2^(2) = 9.41, *p* = 0.009). No between-group differences were found for the remaining categories (family, neighbors, friends, coworkers, public services, nobody).

### Loneliness and Well-Being Changes Over Time

Analysis of the R-UCLA scores over one year showed a significant increase in loneliness levels [*F*(1, 91) = 9.62, *p* = 0.003; t1: 40.76 ± 10.76 vs. t2: 43.43 ± 12.30]. However, no main effect of religious attendance [*F*(2, 91) = 0.08, *p* = 0.922)] or an interaction between religious attendance and time [*F*(2, 91) = 0.28, *p* = 0.757] were found (not in line with H5).

Similarly, GHQ scores increased over the one-year period [*F*(1, 91) = 4.87, *p* = 0.030; t1: 65.88 ± 12.91 vs. t2: 70.21 ± 17.87). However, no main effects of religious attendance [*F*(2, 91) = 1.41, *p* = 0.249], nor an interaction between the factors [*F*(2, 91) = 1.09, *p* = 0.341], were found.

### Discussion

The current analysis aimed to examine the relationships between religious attendance, social network size, loneliness and mental well-being in young Polish adults during the COVID-19 pandemic.

#### Religious Attendance and Social Networks

In line with our expectations (H1), significant between-group differences in the objective characteristics of social networks were observed (i.e., larger social network size was found in both FAs and IAs, compared to NAs). This finding is congruent with previous conceptualizations of religious services as a multilayer social activity that can promote engagement in the various forms of worship-related social bonding (Dunbar, 2021; Rote et al., 2013). The notion that religious attendance may promote social contact with other attendees is reflected in the effect of religious attendance on the number of contacts associated with religious activities in the current study.

Yet, even after subtracting the number of contacts associated with religious worship from the overall social network size score, FAs still had larger social networks compared to NAs (in line with H3). This finding suggests that the effects of religious attendance on social functioning cannot be solely attributed to the additional contacts stemming from worship groups per se, which is in line with previous work emphasizing more general, structural effects that religion can have on one’s social network (Lewis et al., 2013; Ten Kate et al., 2017). More detailed investigation of the twelve different types of social relationships measured via the SNI found no between-group differences in contacts with parents and close friends. Interestingly, the lowest frequency of being in a marital-like relationship was found in the FAs group. Furthermore, in line with these results, the investigation of COVID-19 related social items revealed that FAs were less often planning to spend the subsequent weeks with their partners and they were less often expecting help from a partner in the case of COVID-19 quarantine. Given the negative association between religiosity and intimate coresidential unions (Thornton et al., 1992), these results suggest that FAs were both less likely to be in a marital-like relationship and to live with a romantic partner.

At the same time, analysis of the SNI subscales revealed that FAs had more regular contact with family members other than partners, parents and children, and with students or teachers from school, university, technical training, or adult education. Furthermore, FAs were more likely to spend the subsequent two weeks’ time with their parents and siblings. This finding suggests that FAs in the current study may be more likely to still be living with their family of origin, which in effect may have increased FAs’ propensity for contact with family members other than spouses, parents or children, as measured by the SNI. Furthermore, more participants from the FAs group reported being a student (85% of the group) and currently living in a city large enough (> 500 k inhabitants) to host an academic institution (73%). In both remaining groups, the number of students (NAs: 81%/ IAs: 79%) was more congruent with the percentage of participants living in cities large enough to host academic institutions (NAs: 88%/ IAs: 86%). This discrepancy suggests that FAs may be more likely to study out of their current residence place and to possibly commute for academic purposes. Both theoretical formulations (Tinto, 1993) and empirical data (Benson, 2007) suggest that commuter students could experience more difficulties in effectively forming new friends within the college community compared to non-commuter students. However, the methods of the current study did not distinguish between participants who study in their city of origin and those who moved to live on campus. Thus, even though the commuter students may be overrepresented in the FAs group, the difference in the size of the academic network may still stem from patterns of campus living, especially given the fact that the larger discrepancy between the fraction of the FAs currently living in (62.2%) and originating from the city (> 500 k; 25.0%). Further research will be needed to evaluate whether differences in size of the academic based network between FAs and NAs stem from different patterns of involvement in campus activities or other factors.

#### Religious Attendance, Loneliness and Mental Well-Being

Interestingly, despite the differences in objective social network size, religious attenders did not show lower levels of PSI than NAs (not congruent with H2). Furthermore, similar to (Rote et al., 2013), we observed a significant indirect effect of religious attendance on loneliness via social network size. However, this effect was limited to the FAs vs. NAs, and observed also for the adjusted social network size score, which did not include worship-based affiliates. In line with our hypothesis (H4) that the effects of religious attendance on mental well-being are sequentially mediated by both objective social network characteristics and subjective appraisals of one’s social bonds, this trajectory could also have been further extended towards mental well-being.

The loneliness measurement method used in the current study allows for investigation of the various aspects of PSI, ranging from loneliness in intimate relationships through loneliness in more casual social networks to the lack of a sense of belonging to a larger group or community. Interestingly, we did not observe between-group differences in the Intimate Others score, even though FAs were less often involved in marital-like relationships compared to the other groups (and particularly NAs). A single relationship status has been previously linked in young Polish adults with increased romantic and family loneliness (Adamczyk, 2016). Furthermore, it has been shown that emotional loneliness in young adults is linked only with the presence of a romantic partner, but not with the presence of a non-romantic close other (e.g., close friend), nor general social network size characteristics (Green et al., 2001).

On the other hand, the overall size of the social network (but neither the presence of a partner or close other) was shown to be significantly negatively correlated with social, but not emotional, loneliness in young adults (Green et al., 2001). Thus, the pattern of the between-group findings for the remaining loneliness subscales (lower levels of loneliness associated with more casual social relationships and a general sense of belonging to community in FAs compared to NAs) was congruent with the findings from the objective social networks measurement (increased overall network size, more contacts from further family, academic and church-based networks in FAs compared to NAs). Furthermore, a Polish study on 19–25 year old young adults also found that the effects of remaining single on romantic loneliness are mediated by perceived social support from family, with high support from the family mitigating the negative impact of prolonged single status on romantic loneliness (Adamczyk, 2016). Thus, it is plausible that the effects of an increased likelihood of not being in a marital-like relationship may have been mitigated by larger support networks in FAs. Furthermore, this effect may have suppressed the between-group differences in the overall loneliness score in the current study.

#### Religious Attendance, Loneliness and the COVID-19 Pandemic

Finally, additional evidence that the effects of religious attendance on loneliness and mental well-being cannot be fully explained by worship-related social groups comes from our longitudinal examination. In spite of our hypothesis (H5), we observed that PSI increased while mental well-being decreased over a one-year follow-up time in our participants, and neither of these effects were moderated by religious attendance. As religious ceremonies were among the very few exceptions for public gatherings that were not prohibited in Poland since the start of the COVID-19 restrictions, these findings suggest that being able to participate in social gatherings during worship practices did not translate into decreased loneliness in the religious attenders who completed follow-up in the current study.

## Conclusion

In conclusion, the results of the current study suggest that previous studies that have used aggregate measures of social integration or social network size may have failed to account for some aspects of the social network composition, which may differentiate FAs from NAs. Importantly, while we have replicated previous results linking frequent religious attendance with loneliness and mental well-being via social network size, these effects could still be observed after exclusion of the worship-based groups from the analyzed social networks. This finding adds to previous evidence showing that the link between religion and social functioning does not rely solely on interactions within one’s religious community, and that religious affiliation can have a more nuanced impact on the structure and quality of various social ties. For example, as suggested by the obtained results, this effect may be linked to potential differences in the pattern of independent living or academic activity, which should be subjected to more detailed investigation in future studies.

Furthermore, there are other possible sources of the intergroup differences in social functioning which were not explored in the presented study. For example, it has been shown that religiosity might be positively correlated with different measures of general social affiliation, i.e. a preference for pleasant relationships and interactions (Van Cappellen et al., 2017). The motivation for social affiliation might thus underlie the tendency to bond with various groups of people, which in turn might result in wider social networks. Moreover, another study found that religious involvement is indirectly related to more social ties over time, and this relationship is mediated by more efficient emotion regulation (Semplonius et al., 2015). As those mechanisms could have a significant impact on both perceived social isolation and mental well-being, it would be of great value to explore these links in future examinations of the topic.

Moreover, despite multiple structural differences in social functioning, FAs and NAs did not significantly differ in psychological outcomes (i.e., loneliness and well-being), suggesting that, in a time of crisis, these two groups can rely on distinct aspects of their social networks. While some social characteristics of the FAs group may have constituted protective factors against negative outcomes (e.g., more contact with family members and fellow students), others could have mitigated these effects (e.g., lack of a romantic partner). Thus, similar to previous research, this finding highlights the importance of a more nuanced investigation of the social consequences of religious affiliation and its relation to psychological well-being (Hastings, 2016; Ten Kate et al., 2017).

### Study Limitations

While we have utilized well-established measurement methods to examine objective social network characteristics and loneliness in large groups of young adults, who were followed over a one year period, some significant limitations of the current design should be pointed out. First, our measurement of religious attendance was limited to a single item and we did not collect information on formal membership in specific religions. Given the demographic structure of the Polish population, it is very likely that a prevailing majority of our participants were members of the Roman Catholic Church (Borek et al., 2020). However, without direct confirmation of the religious identification of our participants, no specific mechanisms linked to the particularities of service can be pinpointed in the current study.

Furthermore, we did not investigate more qualitative characteristics of religious involvement in our participants, and factors like spirituality (Hill & Pargament, 2003), non-organizational involvement in religious practices (Anderson & Nunnelley, 2016) or religious coping (De Berardis et al., 2020; Pargament et al., 2011), may be linked to the effects observed in the current study. Similarly, religious coping has been shown to be significantly associated with loneliness and mental well-being during the COVID-19 crisis (Saud et al., 2021; Yıldırım et al., 2021), and was not assessed in the current study.

Furthermore, even though other studies have shown the stability of social networks in the initial months of the COVID-19 pandemic (Bond, 2021), it cannot be excluded that with such a long follow-up period that the participants may have undergone multiple changes in their social network structure, associated both with general factors (e.g., changes in the relationship status over time) and COVID-19 specific issues (e.g., losing a friend or relative due to COVID-19). Yet, to avoid excessive dropout, we decided not to include the SNI in the follow-up assessment, which may be seen as a major limitation of the current study.

Finally, some characteristics of the population investigated in the current study may be seen as limitations. For example, most of our participants were female, and this population was (insignificantly) more pronounced in religious attenders. Finally, only about one-third of our participants filled out the follow-up survey and this group was dominated by NAs.
